# High prevalence of soil-transmitted helminth infections among primary school children, Uttar Pradesh, India, 2015

**DOI:** 10.1186/s40249-017-0354-7

**Published:** 2017-10-09

**Authors:** Sandipan Ganguly, Sharad Barkataki, Sumallya Karmakar, Prerna Sanga, K. Boopathi, K. Kanagasabai, P. Kamaraj, Punam Chowdhury, Rituparna Sarkar, Dibyendu Raj, Leo James, Shanta Dutta, Rakesh Sehgal, Priya Jha, Manoj Murhekar

**Affiliations:** 1 National Institute of Cholera & Enteric Diseases, Indian Council of Medical Research, Kolkata, India; 2 Evidence Action - Deworm the World Initiative, New Delhi, India; 3 GFK Mode, Mumbai, India; 4 Department of Epidemiology, National Institute of Epidemiology, Indian Council of Medical Research, Chennai, India; 5 Department of Medical Parasitology, Postgraduate Institute of Medical Education and Research, Chandigarh, India

**Keywords:** Soil-transmitted helminths, Uttar Pradesh, India

## Abstract

**Background:**

Soil-transmitted helminth (STH) infections often affect the poorest and most deprived communities. In order to generate reliable data for planning a school based deworming program, we conducted a survey among primary school children studying in government schools in the Indian state of Uttar Pradesh. The objectives of our survey were to estimate the prevalence and intensity of STH infections.

**Methods:**

We conducted a cross-sectional survey among children studying in 130 primary schools from 9 agro-climatic zones, during May – August 2015. Information about socio-demographic details, defecation and hand-hygiene practices, and stool samples were collected from the school children. Stool samples were examined using the Kato-Katz method.

**Results:**

Stool samples from 6421 school children were examined. The overall weighted prevalence of any STH in the State was 75.6% (95% *CI*: 71.2–79.5). The prevalence was more than 50% in six of the nine agro-climatic zones. *A. lumbricoides* was the most prevalent STH (prevalence: 69.6%), followed by hookworm (prevalence: 22.6%) and *T. trichura* (4.6%). The majority of the STH infections were of low intensity. The practice of open defecation and not washing hands with soap after defecation and residence in kutcha house were significant risk factors of STH infection.

**Conclusions:**

STH prevalence among primary school children in Uttar Pradesh was high. Given the WHO guidelines on deworming frequency according to STH prevalence, Govt of Uttar Pradesh needs to implement a school-based deworming program with bi-annual frequency. The findings of our survey would also help monitor the performance of school based deworming programme.

**Electronic supplementary material:**

The online version of this article (10.1186/s40249-017-0354-7) contains supplementary material, which is available to authorized users.

## Multilingual abstracts

Please see Additional file 1 for translations of the abstract into the five official working languages of the United Nations.

## Background

Infection with four species of nematodes – the roundworm (*Ascaris lumbricoides*), the whipworm (*Trichuris trichiura*), and the hookworms (*Necator americanus* and *Ancylostoma duodenale*), collectively referred to as soil-transmitted helminths (STH), are among the most common neglected tropical diseases worldwide [1]. STH are widely distributed in tropical and subtropical areas with warm and moist climates, and are more prevalent where sanitation and hygiene of the population is poor. The World Health Organization (WHO) estimates that 880 million children are at risk of STH infection worldwide and require treatment [2], of which 241 million are in India [3]. The greatest numbers of STH infections occur in sub-Saharan Africa, East Asia, China, India and South America [4, 5].

STH infections cause morbidity by adversely affecting nutritional status and impairing cognitive processes. A number of studies have suggested that STH infection may result in delayed physical growth and impaired cognitive development, particularly among school aged children [4–9]. STH infections are considered a leading cause of sickness, school absenteeism, and disability adjusted life years (DALYs) lost [1, 10].

WHO recommends mass deworming for control of STH infection [1]. Annual mass deworming is intended for high-risk groups such as pre-school and school aged children. In school-based deworming, administration of anthelminthic drugs to school-aged children is done through the existing school infrastructure, because it is simple, safe, cost effective, scalable, and provides a platform to reach high-risk populations. Despite reinfection, periodic drug treatment reduces the number of heavy infections in the community, reduces environmental contamination and risk of infection for other people, reduces micronutrient loss (e.g. iron loss through intestinal bleeding in hookworm infection), and improves nutritional status, cognitive functions and learning abilities [1].

Although periodic treatment with anthelminthic for the control of intestinal parasitic infection is highly effective and inexpensive, a careful study of epidemiology of STHs is needed to guide the deworming frequency [1]. The Indian State of Uttar Pradesh did not have a statewide school-based deworming programme prior to 2015. The government of Uttar Pradesh began planning a statewide school-based deworming programme in 2015, but had limited information about the prevalence and intensity of STH infections in the State. Against this background, we conducted a survey among school children studying in government primary schools in Uttar Pradesh, in order to generate estimates of the current statewide prevalence and intensity of STH infections and guide the deworming programme in the state.

## Methods

### Ethics, consent and permission

The Institutional Ethics Committee of the ICMR - National Institute of Epidemiology, Chennai, approved the study protocol. Written informed consent from parents of all students assenting to participate in the study was obtained prior to the interviews. All schools surveyed were provided with deworming medication. Permission to conduct the survey in schools was obtained from the Departments of Health and Education, Government of Uttar Pradesh.

### Study setting

Uttar Pradesh is one of the most populated states with a population of 199 million residing in 71 districts, as per the 2011 census. The State is divided into 9 agro-climatic zones, based on soil type, average annual rainfall, and temperature [11]. Although there had been no school-based deworming programme in the State prior to 2015, 51 of the 71 districts were endemic for lymphatic filariasis (LF) and annual mass deworming (LF-MDA) with diethycarbamazine (DEC) and albendazole had been conducted in these districts as a part of the National Filaria Control Programme [12].

### Sample size

As the overall goal was to establish a system for periodic collection of parasitological data to monitor school based deworming programme, we used the WHO recommended sentinel school approach for selecting schools for the survey [1]. As per the 2011 census, there were 31,408,995 children aged 5–10 years in Uttar Pradesh. Assuming 1 sentinel school for 250,000 children, we required 126 schools (rounded to 130) to estimate the prevalence of STH in the state and monitor the performance of deworming programme. With 50 children per school [1], the minimum sample size required for estimation of baseline prevalence was 6500. This sample size was inflated by 20% to 7800 to account for a non-response.

### Sampling procedure

We selected 130 schools from 9 agro-climatic zones; the number of schools in each zone was proportionate to the percentage of the 5–10 year old population in each of the zones. We used a two-stage sampling procedure for selecting sentinel schools. In the first stage, we selected 27 districts randomly from the 71 districts in the state. This included 17 LF endemic districts (Additional file 2: Table S1). In the second stage, all the primary schools of the districts selected from each zone were line-listed. The required number of schools for each zone was then randomly selected from the list of schools in the selected districts. To select the required number of children for the survey from the selected school, a random number (between 1 to 5) was assigned to each school. The field team started the survey from the class (grade) corresponding to the random number assigned for the school and enumerated children present in the class (grade) starting from roll number one. If the number of children in the selected class was <60, children from the next class were selected. This procedure was continued till 60 children from each school were selected.

### Data collection

The survey was conducted during May – August 2015. The trained survey teams visited the assigned schools, met the Principal/Head of the school and informed him/her about the objectives of the survey. After obtaining the permission, teams collected information about drinking water and sanitation facilities in the school. The field teams then enrolled 60 children from the selected class and collected their contact details. The survey teams then visited the houses of children enrolled in schools. After obtaining written informed consent from parents/head of the household, respondents (mainly the mothers) for these children were interviewed to collect information about socio-demographic details, house type, source of drinking water, presence of toilet facilities and their use, hand washing practices and history of deworming.

### Collection and transport of stool samples

The teams explained stool sample collection procedure to the child and his/her parents/respondents and provided them with a sample collection kit. Children were asked to defecate on the cardboard sheet the next morning, collect about two teaspoons full stool and place it in the container, screw the cap tightly to prevent leakage and place the container in a zip-lock bag. The field teams collected stool samples in the morning from the houses of children. The stool samples were transported in cool boxes to the field laboratory within 3–4 h of sample collection. In the laboratory the samples were kept in cool boxes with ice packs until processed.

### Processing of samples

The field laboratory was setup in the district hospitals and each lab team consisted of two parasitologists and one-laboratory technician. The samples were processed using a kit based on the double Kato Katz method (Vestergaard Frandsen, New Delhi) following the manufacturer’s instructions [13]. For each sample two slides were prepared and were independently examined by two parasitologists. The intensity of STH was measured as eggs per gram of stool, and was classified according to WHO guidelines [1].

### Quality control

As a part of quality control, 10% of the slides were double read by exchange between the parasitologists. An independent expert also reviewed the laboratory and survey procedures.

### Data analysis

The data was analysed using survey data analysis module of STATA software (version 13) to estimate the prevalence and intensity of STH. All the estimates were weighted to account for unequal selection probabilities. Chi square test was used to compare proportions. A Geographic Information System (GIS) based spatial interpolation method – Inverse Distance Weighting method (IDW) was used for predicting the prevalence of STH in the state using the prevalence data observed in the surveyed districts. The locations of the schools surveyed along with the un-weighted prevalence of STH infection were integrated into the GIS. ArcGIS version 10 (ESRI, Redlands, CA, USA) was used for mapping. Using the survey data analysis module in STATA, we conducted a multiple logistic regression analysis to identify risk factors associated with STH infection. Odds ratios and 95% confidence intervals were estimated. Variables with *P* value of <0.2 on univariate analysis were included in the multiple logistic regression model.

## Results

The survey was conducted in 130 schools from 27 districts of 9 agro-climatic zones. The majority (93%) of the schools were from rural areas, had a source of drinking water (95%) and had toilet facilities (89%) within the school premises. A total of 7547 children from 130 schools were enrolled and stool samples were collected from 6924 (92%) children. Stool samples from 503 children were rejected for reasons such as inadequate sample, or the sample was mixed with urine/water/soil etc. Samples from 6421 (against the target of 6500, 98.8%) children were analysed to estimate the prevalence of STH in the state.

### General characteristics of children surveyed

The median age of children surveyed was 8 years (IQR: 7–10), 50% were girls and 41% belonged to scheduled caste/tribe. Fathers of 42% of the children, and mothers of 78% of the children had no formal education. Most (97%) of the households had piped water supply and more than three fourth of the households had no latrines and practiced open field defecation (Table 1).

**Table 1:** Socio-demographic characteristics of children surveyed, Uttar Pradesh, 2014 (*n* = 6421)

Characteristics	Number	%
Age (years)		
≤ 6^a^	1160	18.1
7–10	4455	69.4
> 10	806	12.6
Gender		
Boys	3212	50.0
Girls	3209	50.0
Caste		
General / Other backward class	3761	58.6
Scheduled caste/scheduled tribe	2622	40.8
Not disclosed	38	0.6
Religion		
Hindu	5211	81.16
Muslim	1200	18.69
Others	10	0.16
Education of father		
No education	2663	41.5
Primary/middle school	2474	38.5
Secondary school or above	1235	19.2
Not mentioned	49	0.8
Education of mother		
No education	5007	78.0
Primary/middle school	1091	17.0
Secondary school or above	301	4.7
Not mentoned	22	0.3
Occupation of father		
Wage Labourer	3471	54.1
Agriculture/animal husbandry/allied activity	1699	26.5
Self employed/service	1086	16.9
Others	107	0.2
Unemployed	58	0.9
Place of defecation		
Open field	4916	76.6
Latrine	1505	23.4
Source of drinking water		
Public tap/Piped water	6197	96.5
Unprotected dug well or spring	84	1.3
Protected well	74	1.2
Others	66	1.0
Type of house		
Kuccha wall and roof (house made of mud)	1654	25.8
Pucca (Pucca wall and roof) (house made of of concrete, brick and cement)	2789	43.4
Semi Pucca	1978	30.8
Regular handwashing after defecation		
No	78	1.2
With only water	795	12.4
With mud or ash	2323	36.2
With soap	3225	50.2
Child took deworming medicine for LF in last 1 year		
Yes	969	15.1
No	5207	81.1
Don’t know	245	3.8

*(* ^*a*^ *20 children were aged 4 years)*

### Prevalence of soil-transmitted helminths

**Fig. 1:**
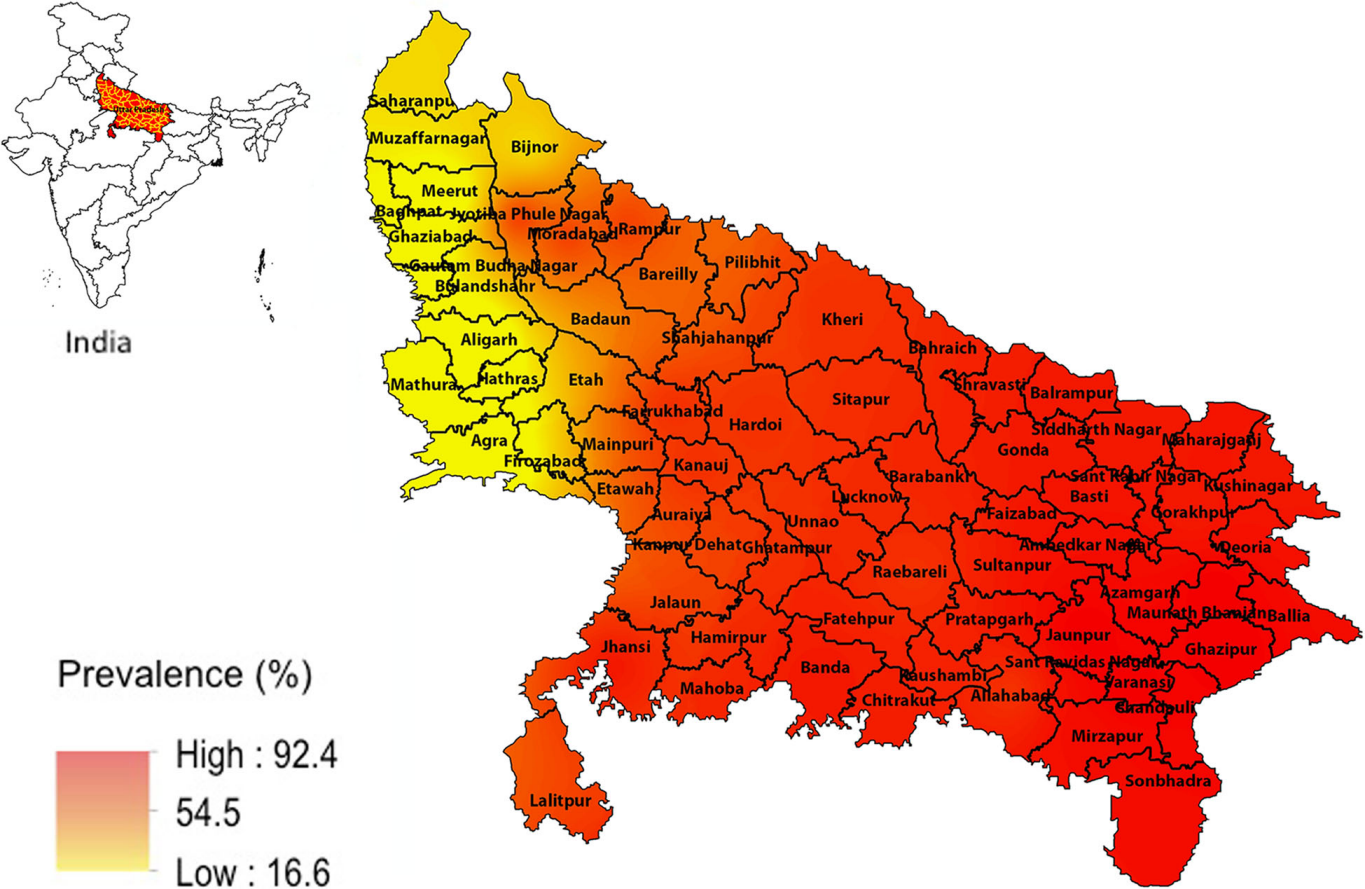
Predicted prevalence map of soil-transmitted helminths, Uttar Pradesh, India, 2015

Of the 6421 children whose stool samples were examined, 4578 had one or more STH infection with a weighted prevalence of 75.6% (71.2–79.5). The prevalence of any STH was more than 50% in six of the nine agro-climatic zones (Fig. 1). In the remaining two Zones (Bhabhar and Tarai, and Western Plain zones), the prevalence ranged between 20 and 50% while in one zone the prevalence was less than 20% (South Western Semi Arid Zone) (Table 2).

**Table 2:** Prevalence of soil-transmitted helminth infections in different agro-climatic zones, Uttar Pradesh, 2014 (*n* = 6421)

Agro-climatic Zone	No. examined	*A. lumbricoides*		*T. trichura*		Hookworm		Any STH	
		No. positive	% (95% *CI*)	No. positive	% (95% *CI*)	No. positive	% (95% *CI*)	No. positive	% (95% *CI*)
Bhabhar and Tarai	299	65	21.7 (11.5–37.2)	7	2.3 (1.3–4.2)	74	24.7 (15.5–37.0)	118	39.5 (27.8–52.5)
Bundelkhand	290	244	84.1 (77.4–89.2)	0	-	60	20.7 (13.4–30.5)	249	85.9 (78.8–90.8)
Central	1660	1257	75.7 (68.9–81.4)	1	0.1 (0–0.4)	428	25.8 (20.4–32.0)	1332	80.2 (74.8–84.7)
Eastern Plain	1167	989	84.7 (78.1–89.6)	268	23.0 (13.2–36.9)	285	24.4 (18.5–31.5)	1078	92.4 (88.0–95.2)
Mid Western Plain	752	546	72.6 (64.4–79.5)	7	0.9 (0.4–2.0)	138	18.4 (11.3–28.3)	568	75.5 (68.4–81.5)
North Eastern Plain	1031	860	83.4 (78.3–87.5)	12	1.2 (0.3–4.2)	235	22.8 (17.1–29.7)	883	85.6 (80.6–89.6)
South Western Semi Arid Zone	542	10	1.8 (0.8–4.3)	5	0.9 (0.3–2.9)	84	15.5 (8.0–27.5)	90	16.6 (8.7–29.3)
Vindhyan Zone	154	136	88.3 (84.2–91.5)	0	-	15	9.7 (4.3–20.6)	137	89.0 (86.1–91.3)
Western Plain Zone	526	27	5.1 (2.5–10.4)	7	1.3 (0.4–4.7)	95	18.1 (13.3–24.2)	123	23.4 (17.9–29.9)
Total	6421	4134	69.6 (64.6–74.3)	307	4.6 (2.6–8.2)	1414	22.6 (19.9–25.7)	4578	75.6(71.2–79.5)

In all the nine agro-climatic zones, *A. lumbricoides* was the most common STH infection with a weighted prevalence of 69.6% (95% *CI*: 64.6–74.3). The prevalence of hookworm and *T. trichiura* infections was 22.6% (95% *CI*: 19.9–25.7) and 4.6% (95% *CI*: 2.6–8.2) respectively (Table 2). The majority (4206, 91.9%) of the children were infected with one STH, while 8.1% (*n* = 369) and 3 (0.07%) were infected with two and three STH respectively.

The weighted prevalence of any STH in the state was 73.1 (95% *CI*: 67.6–78.0) among children aged 5–6 years, 75.6% (95% *CI*: 71.1–79.7) among children aged 7–10 years and 78.8% (95% *CI*: 73.0–83.7) among children aged 11–15 years. The prevalence of any STH was not different by age group or sex [boys: 75.5% (95% *CI*: 71.2–79.3), girls: 75.7% (95% *CI*: 70.9–80.0)] (Table 3).

**Table 3:** Prevalence^a^ (%) of Soil-transmitted helminth infections among children by age group and sex, Uttar Pradesh, 2015

Characteristics	Number of children	Any STH		*A. lumbricoides*		*T. trichura*		Hookwrom	
		Number positive	Prevalence^a^ (95% CI)	Number positive	Prevalence^a^ (95% CI)	Number positive	Prevalence^a^ (95% CI)	Number positive	Prevalence^a^ (95% CI)
Age group (years)									
≤ 6	1160	811	73.1 (67.6–78.0)	751	68.4 (62.6–73.8)	57	4.8 (2.5–8.9)	188	16.8 (13.6–20.5)
7–10	4455	3167	75.6 (71.1–79.7)	2850	69.4 (64.1–74.3)	195	4.2 (2.3–7.7)	1009	23.3 (20.4–26.4)
11–15	806	600	78.8 (73.0–83.7)	533	72.4 (65.4–78.5)	55	6.5 (3.3–12.5)	217	27.3 (22.1–33.2)
Sex									
Boys	3212	2297	75.5 (71.2–79.3)	2040	68.6 (63.3–73.5)	143	4.3 (2.4–7.4)	760	24.3 (21.2–27.6)
Girls	3209	2281	75.7 (70.9–80.0)	2094	70.7 (65.4–75.5)	164	5.0 (2.7–9.1)	654	21.0 (18.1–24.2)

^a^W eighted

### Intensity of STH infection

Majority of the STH infection were of light intensity; only 14 and 12 children had heavy intensity of hookworm and *A. lumbricoides* infections respectively. The mean number of eggs per gram of stool of STH infected person was 1803 for *A. lumbricoides*, 95 for *T. trichiura* and 416 for hookworm (Table 4).

**Table 4:** Intensity of Soil-transmitted helminth infections among children, Uttar Pradesh, 2015

	Negatives (%)	Light (%)	Moderate (%)	Heavy (%)	Mean eggs per gram of stool^a^
*A. lumbricoides*	2287 (35.6)	3829 (59.6)	293 (4.6)	12 (0.2)	1803
*T. trichura*	6114 (95.2)	307 (4.8)	0 (0)	0 (0.0)	95
Hookworm	5007 (78.0)	1361 (21.2)	39 (0.6)	14 (0.2)	416

(^a^Among infected children)

**Table 5:** Risk factors associated with soil-transmitted helminth infection, Uttar Pradesh, India, 2015

Variables		Number Infected (*n* = 4578)	Number Uninfected (*n* = 1843)	Unadjusted *OR* (95% *CI*)	*P*-value	Adjusted *OR* (95% *CI*)
Gender	Male	2297	915	1.0 (0.9–1.2)	0.857	
	Female	928	2281	1		
Age	> 8 years	2105	812	1	0.155	1.2 (0.99–1.4)
	≤ 8 years	2473	1031	1.1 (0.9–1.3)		
Caste	SC/ST	1831	791	0.9 (0.7–1.2)	0.468	
	Others	2739	1022	1		
Mother’s education	Illiterate	3549	1458	0.9 (0.7–1.1)	0.182	1.2 (0.9–1.6)
	Literate	1019	373	1		
Type of house	Kutcha	1290	364	1.4 (1.1–8)	0.008	1.3 (1.1–1.5)
	Pucca/Semi -Pucca	3288	1479	1		
Place of defecation	Open field	3687	1229	2.0 (1.4–2.7)	0.000	1.8 (1.3–2.4)
	Latrine	891	614	1		
Received deworming medicine in last year	No	3746	1461	1.0 (0.8–1.4)	0.774	
	Yes	664	305			
Handwashing with soap after defecation	No	2463	733	1.5 (1.2–2.0)	0.001	1.4 (1.1–1.8)
	Yes	2115	1110			

### STH prevalence by LF MDA

Of the 27 districts selected for STH survey, 17 were endemic for lymphatic filariasis, where LF-MDAs had previously been conducted. The un-weighted prevalence of STH infection in the 17 districts covered under the LF-MDA was significantly higher (3748/4558, 82.3%) as compared to the 10 districts not endemic for LF (830/1863, 44.5%, *P* < 0.001).

### Risk factors associated with soil-transmitted helminths

Children who reported that they defecated in open fields and children who did not wash hands with soap after defecation were more likely to have one or more STH infection. Children residing in kutcha houses (houses made of mud) were 1.3 (95% CI: 1.1-1.5) times more likely to have STH infection, as compared to those residing in pucca (house made of concrete, brick and cement) or semi- Pucca houses. (Table 5).

## Discussion

The present survey was conducted to generate reliable estimates of STH prevalence in the state of Uttar Pradesh in order to provide data to plan the school-based deworming programme. The overall prevalence of any STH infection among children studying in government primary schools in the state was very high, with three out of four children attending government primary schools found to have STH infections. The prevalence was high in entire State, except few districts in western Uttar Pradesh. *A. lumbricoides* and hookworm were the most common STH. The practices of open field defecation and not washing hands with soap after defecation were significantly associated with STH infection.

There are very few published reports on the prevalence of STH in the Uttar Pradesh. A study conducted in few villages in Shahjahanpur district in 1994 reported 29.2% prevalence of intestinal parasitoses, while another study conducted in seven villages in Kanpur district in 2003 reported a prevalence of hookworm infection of 34% [14, 15]. Other studies among school-going children in Ghaziabad and Barielly showed low prevalence (< 10%) of STH; however these studies did not use the WHO recommended Kato-Katz method for stool examination [16–18]. Our survey provides most comprehensive information about STH prevalence and intensity in the State of Uttar Pradesh. The low prevalence of trichuris infection observed in our study is consistent with earlier published reports [18, 19].

The STH prevalence in our study was found to be high in all age groups; with 73% of children aged 5–6 years positive for any STH. This indicates that STH infections are acquired during children’s pre-school years. A study by Awasthi et al. among pre-school children in rural villages of Sant Ravidas Nagar district in Uttar Pradesh indicated a very high prevalence (65.9%) of geo-helminths treatable by albendazole [20]. The transmission of STH is facilitated by the widespread practice of open defecation prevalent in the State. The findings of the national level survey on cleanliness and sanitation conducted in 2015 indicated that only 29.5% of the households in rural Uttar Pradesh had sanitary toilets and 92.4% people in the households having toilets, were using them [21].

In Uttar Pradesh, as a part of the LF-MDA albendazole has been administered in the LF endemic districts since 2004. The previous round of the LF-MDA was conducted during 2014. The reported coverage has been more than 80% in all years except 2013, when the coverage fell to 71% [12]. Despite the high reported coverage, STH prevalence was found to be high, and in excess of 80% in most areas. Although re-infections with STH are known to occur after mass deworming and the prevalence tends to return to pre-treatment level, periodic deworming in the long run is expected to reduce the prevalence by reducing the worm load and reducing environmental contamination and risk of infection for other people [1, 22]. Very high prevalence of STH in most of the zones in Uttar Pradesh, despite LF-MDAs probably suggests its limited impact in the State. The reported figures of coverage are generally high, but information about validated coverage of LF-MDA, and more importantly compliance, is necessary to make reliable conclusions about the impact.

In three agro-climatic zones, the STH infection was found to be low. None of the seven districts from these zones were covered under LF-MDA. The lower prevalence in these zones could be on account of agro-climatic conditions as well as distribution of risk factors that were found to be significantly associated with STH infection. The proportion of households having latrines was 18.3% (923/5054) and 42.6% (582/1367) respectively, in the six zones with high STH prevalence and three zones with low prevalence (*P* < 0.001). The corresponding proportions for hand washing with soap after defecation was 71.3% and 44.5% respectively.

The current WHO guidelines for population-based treatment for STH infections focus on the treatment of school-aged children between the ages of 5 to 14 years [1]. The guidelines also suggest that other at-risk groups such as preschool age children may also benefit from treatment [23]. There has been a debate about the impact of school-based deworming programmes on health and educational outcomes, with some reviews indicating limited or no effects [24, 25]. In addition, the results of mathematical models as well as meta-analysis suggest that expanding deworming programmes community-wide is likely to interrupt the transmission and reduce the prevalence of STH in the high-risk group of school-aged children [26, 27]. Studies also indicate that improvements in sanitation were associated with reduced risk of transmission [28]. School-based health hygiene education intervention was also found to be effective in increasing STH knowledge and in reducing STH infection [28]. The programme managers in Uttar Pradesh need to consider the available evidence while designing a comprehensive control programme in the State.

Our study had certain limitations: First, we did not include private schools in the survey, neither did we attempt to cover children who had dropped out of school. Focusing the survey only in public schools might have over-estimated the actual prevalence of STH infection among school children in the state. Second, although WHO recommends the use of the Kato Katz method for STH prevalence surveys [29], the technique is known to have low sensitivity as a diagnostic test especially for low intensity infections [30, 31]. Kato Katz method requires taking only one day’s sample rather than consecutive days as egg excretion is known to fluctuate [31]. The actual prevalence of infection could therefore also be higher than observed in our study. Third, our study focused on three major STH commonly reported in India. We did not make any attempt to examine for *Strongyloides stercoralis*, another STH. Fourth, our survey was designed to estimate the prevalence at the level of agro-climatic zone and not to compare the STH prevalence among LF and non-LF districts. It was therefore not possible to estimate weighted prevalence for LF and non-LF districts.

## Conclusions

STH prevalence among primary school children in Uttar Pradesh was high. WHO recommends annual treatment in areas where STH prevalence is between 20% and 50%, and a bi-annual treatment in areas with prevalence rates of over 50% [3]. In view of high prevalence of STH infection, we recommended that the Government of Uttar Pradesh initiate a school-based deworming programme with bi-annual frequency. These two rounds of deworming would be in addition to the LF-MDA ongoing in 51 districts. The school-based deworming could also be supported by hygiene education intervention, as indicated by the association between hygiene practices and STH in this and other studies [28]. Further, given the high prevalence of STH in all age groups, expanding the deworming programme to pre-school children as well as other age-groups may further reduce the load of infection in community. It is also necessary to carefully document the coverage and compliance to the deworming programmes including the LF-MDA. As a long-term solution for control STH infection, it is also necessary to improve the sanitation levels in the area, as the majority of the houses did not have latrines and most of the children were defecating in open fields. Provision of latrines, may, to a great extent, deter open defecation and help in limiting spread of these infections.

## Additional files

Additional file 1: Multilingual abstracts in the five official working languages of the United Nations. (PDF 746 kb)

Additional file 2: Table S1. Agro-climatic zones of Uttar Pradesh and districts sampled. (DOC 37 kb)

## Abbreviations

LF: Lymphatic filariasis
MDA: Mass drug administration
STH: Soil-transmitted helminths
WHO: World Health Organization
