# Supplementary material for: High prevalence of soil-transmitted helminth infections among primary school children, Uttar Pradesh, India, 2015
Source: Infect Dis Poverty. 2017 Oct 9;6:139. doi: 10.1186/s40249-017-0354-7 (PMC5632835; doi:10.1186/s40249-017-0354-7)
Supplement: Supplementary file 1 — Multilingual abstracts in the five official working languages of the United Nations. (PDF 746 kb) [file 40249_2017_354_MOESM1_ESM.pdf]

Translation of the abstract into the five official working languages of the United Nations

ارتفاع معدل انتشار عدوى الديدان الطفيلية المنقولة بالتربة بين أطفال المدارس الابتدائية، أُتّر برديش، الهند، 2015

ساندیبان غانغولي، شاراد برکاتکي، سوماليا كارماكار، بريرنا سانغا، ك. بوباثي، ك. كاناجاساباي، ب. كاماراج، بونام شودري، ريتوبارنا ساركار، ديبيندو راج، ليو جيمس، شانتا دوتا، راكيش سهغال، بريا جا، مانوج مورهيكار

ملخص
خلفية: <bx/> عدوى الديدان الطفيلية المنقولة بالتربة (STH) غالبا ما يكون تأثيرها على المجتمعات المحلية الأفقر والأكثر حرمانا. سعيا إلى توليد بيانات موثوقة بغرض تخطيط برنامج لطرد الديدان قائم على المدرسة، أجرينا استطلاعا بين أطفال المدارس الابتدائية الذين يدرسون في المدارس الحكومية بولاية أُتّر برديش الهندية. كانت أهداف استطلاعنا تقدير معدل انتشار وكثافة عدوى الديدان الطفيلية المنقولة بالتربة.
الوسائل: <bx/> أجرينا دراسة مقطعية بين الأطفال الدارسين في 130 مدرسة ابتدائية من تسع مناطق ذات مناخ زراعي، في الفترة بين مايو - أغسطس 2015. تم جمع معلومات عن التفاصيل الاجتماعية الديموغرافية، والتغوط، وعادات نظافة اليد، وعينات براز من أطفال المدارس. تم فحص عينات البراز باستخدام تقنية كاتو-كاتز.
النتائج: <bx/> تم فحص عينات براز لعدد 6421 من أطفال المدارس. معدل الانتشار المرجح لأي عدوى ديدان طفيلية منقولة بالتربة في الدولة كان 75.6 (95٪*فاصل الثقة*: 71.2 - 79.5). معدل الانتشار هنا كان أكثر من 50٪ في ست مناطق من المناطق التسع ذات المناخ الزراعي. *دودة الصَّفَرُ الخَراطينِيّ (أ.* لومبريكويدس) *()* كانت عدوى الديدان الطفيلية المنقولة بالتربة الأكثر انتشارا (معدل الانتشار: 69.6٪)، تليه الدُّودَةُ الشِّصِيَّة (معدل الانتشار: 22.6٪) و *المسلكة* الشعرية الذيل) ت. تريكو (4.6%). غالبية الإصابات بعدوى الديدان الطفيلية المنقولة بالتربة كانت منخفضة الكثافة. كانت عادة التغوط في العراء وعدم غسل اليدين بالصابون بعد التغوط والإقامة في بيوت كوتشا، عوامل خطر بارزة مقترنة بالإصابة بعدوى الديدان الطفيلية المنقولة بالتربة.
الاستنتاجات: <bx/> معدل انتشار عدوى الديدان الطفيلية المنقولة بالتربة بين أطفال المدارس الابتدائية في ولاية أُتّر برديش كان مرتفعا. وبالنظر إلى المبادئ التوجيهية لمنظمة الصحة العالمية بشأن تكرر طرد الديدان مجاراة لانتشار عدوى الديدان الطفيلية المنقولة بالتربة، فإن حكومة ولاية أُتّر برديش تحتاج إلى تنفيذ برنامج طرد ديدان قائم على المدرسة يتكرر مرتين في السنة. نتائج استقصاءنا أيضا ستساعد على رصد أداء برنامج طرد الديدان قائم على المدرسة.

Translated from English version into Arabic by Mona Fahmy, through

TRANSLATORS
WITHOUT BORDERS

2015 年印度北方邦地区小学生土源性线虫高感染率

Sandipan Ganguly, Sharad Barkataki, Sumallya Karmakar, Prerna Sanga, K. Boopathi, K. Kanagasabai, P. Kamaraj, Punam Chowdhury, Rituparna Sarkar, Dibyendu Raj, Leo James, Shanta Dutta, Rakesh Sehgal, Priya Jha and Manoj Murhekar

摘要：
引言：土源性线虫（STH）感染通常影响最贫困的社区。为了得到可靠的数据在学校进行驱虫计划。我们对印度北方邦公办小学的小学生进行了一项调查。此次调查的目的是估计土源性线虫的感染率和感染强度。
方法： 2015 年 5 月-8 月，对 9 个农业气候区的 130 所小学的小学生进行横断面调查。收集小学生的社会人口统计信息，排便和洗手的卫生习惯以及粪便样本。使用 Kato-Katz 方法检查粪样。
结果：共检查 6 421 名小学生的粪样。该地区土源性线虫总体加权感染率为 75.6%（95％*CI*：71.2 - 79.5）。9 个农业气候区域中的 6 个地区感染率超过 50％。感染率最高的是蛔虫（69.6%），其次是钩虫（22.6%）和人鞭虫（4.6%）。大多数土源性线虫感染强度较低。露天排便、排便后不使用肥皂洗手和居住在 kutcha 房屋是与土源性线虫感染相关的主要危险因素。
结论：印度北方邦小学生土源性线虫感染率居高不下。根据世界卫生组织土源性线虫的驱虫指南，北方邦政府需要制定一个以学校为基础的两年一次的驱虫计划。本调查研究结果也有

助于监测基于校园的驱虫计划。

Translated from English version into Chinese by Lei Sun, edited by Pin Yang

Forte prévalence des helminthiases transmises par le sol parmi les enfants des écoles primaires dans l'Uttar Pradesh, Inde, 2015

Sandipan Ganguly, Sharad Barkataki, Sumallya Karmakar, Prerna Sanga, K. Boopathi, K. Kanagasabai, P. Kamaraj, Punam Chowdhury, Rituparna Sarkar, Dibyendu Raj, Leo James, Shanta Dutta, Rakesh Sehgal, Priya Jha, Manoj Murhekar

Résumé

Contexte : Les géohelminthiases souvent les populations les plus pauvres et démunies. Afin d'obtenir des données fiables pour planifier un programme de vermifugation à l'école, nous avons mené une étude parmi les enfants des écoles primaires publiques de l'état indien d'Uttar Pradesh. Les objectifs de notre étude étaient d'estimer la prévalence et l'intensité des géohelminthiases.

Méthodes : Nous avons mené une étude transversale parmi des enfants scolarisés dans 130 écoles primaires de 9 zones agro-climatiques, entre mai et août 2015. Des informations sur la situation sociodémographique et les pratiques concernant la défécation et l'hygiène des mains ainsi que des échantillons de selles ont été recueillis auprès des écoliers. Les échantillons de selles ont été examinés à l'aide de la méthode de Kato-Katz.

Résultats : Les échantillons de selles de 6421 écoliers ont été examinés. La prévalence pondérée totale des goéhelminthiases dans l'état était de 75,6 (IC à 95 % : de 71,2 à 79,5). La prévalence était de plus de 50 % dans six des neuf zones agroclimatiques. *Ascaris lumbricoides* (*A. lumbricoides*) était le géohelminthe le plus prévalent (prévalence : 69,6 %), suivi des ankylostomes (prévalence : 22,6 %) et de *Trichuris trichura* (*T. trichura*) (4,6 %). La majorité des géohelminthiases étaient de faible intensité. La pratique de la défécation en plein champ, l'absence de lavage des mains avec du savon après avoir déféqué et la résidence dans des huttes au sol en terre étaient des facteurs de risque de géohelminthiase significatifs.

Conclusions : La prévalence des HTS parmi les élèves des écoles primaires de l'Uttar Pradesh était élevée. Compte tenu des directives de l'OMS concernant la fréquence de vermifugation en fonction de la prévalence des HTS, le gouvernement de l'Uttar Pradesh doit mettre en place un programme de vermifugation bisannuelle dans les écoles. Les résultats de notre étude pourraient également aider à contrôler les résultats de ces programmes.

Translated from English version into French by Suzanne Assenat, through

Высокая распространенность заражения гельмитами среди детей младшего школьного возраста. Уттар-Прадеш, Индия, 2005 год.

Сандипан Гангули, Шарад Баркатаки, Сумалья Кармакар, Прнна Санга, К. Боопати, К. Канагасабай, П. Камарай, Пунам Чоудхури, Ритуперна Саркар, Дибенду Радж, Лев Джеймс, Шанта Дутта, Ракеш Сегал, Прия Джа, Маной Мурхекар

Аннотация

Сведения для справки: Заражение гельминтозами передаваемыми через почву (ГПП)

зачастую затрагивает самые бедные и обездоленные сообщества. Для получения достоверных данных, для введения программы дегеминтизации в школах, мы провели опрос среди учеников начальных школ, учащихся в государственный школах в Индийском штате Уттар-Прадеш. Цель нашего опроса - оценка распространенности и интенсивности заражений ГПП.
Методы: В мае-августе 2015 года мы провелиперекрестное обследование среди детей, обучающихся в 130 начальных школах из девяти агро-климатических зон. Информация о социально-демографических подробностях, о навыках дефекации и ручной гигиены, а также образцы стула были собраны у школьников. Образцы стула были исследованы при использовании метода Като-Каца.
Результаты: Были изучены образцы стула от 6 421 школьников. Общая взвешенная распространенность любого из видов ГПП в государстве составляла 75,6 (95% *CI*: 71,2 - 79,5). В шести из девяти агро-климатических зон распространенность составила более 50%. *Ascaris lumbricoides* (*A. lumbricoides*) - наиболее распространенный ГПП(распространенность: 69,6%), за которым следовали анкилостома (распространенность: 22,6%) и (*Trichuris trichura*) *T. трихура* (4,6%). Большинство инфекций ГПП имели низкую интенсивность. Практика открытой дефекации без последующего мытья рук мылом и проживания в домах типа кутч (из высушенного на солнце кирпича) были значимыми факторами риска, связанными с инфекцией ГПП.
Выводы: Уровень распространенности ГПП среди детей младшего школьного возраста в штате Уттар-Прадеш был высоким. Учитывая принципы ВОЗ, в отношении частоты дегельминтизации в соответствии с распространенностью ГПП, правительству Уттар-Прадеша необходимо внедрить программу дегельминтизации в школы с проведением программы каждые полгода. Результаты нашего опроса также помогут контролировать эффективность программы дегельминтизации в школе.

Translated from English version into Russian by margarita, through

TRANSLATORS
WITHOUT BORDERS

Alta prevalencia de infecciones por helmintiasis transmitidas por el suelo entre niños de colegios primarios, Uttar Pradesh, India, 2015

Sandipan Ganguly, Sharad Barkataki Sumallya Karmakar, Prerna Sanga, K. Boopathi, K. Kanagasabai, P. Kamaraj, Punam Chowdhury, Rituparna Sarkar, Dibyendu Raj, Leo James, Shanta Dutta, Rakesh Sehgal, Priya JAI, Manoj Murhekar

Resumen
Antecedentes: Las infecciones por helmintiasis transmitidas por el suelo (STH, en inglés) a menudo afectan a las comunidades más pobres y necesitadas. Con el fin de generar datos confiables para la planificación de un programa de desparasitación escolar, se realizó un estudio a niños de primaria que estudian en colegios públicos del estado indio de Uttar Pradesh. Los objetivos del estudio se enfocaron en la evaluación de la prevalencia y en la intensidad de las infecciones por STH.
Métodos: Se realizó un estudio transversal, desde mayo hasta agosto de 2015, a niños de 130 colegios primarios de nueve zonas agroclimáticas. Se recolectó información sobre datos sociodemográficos, defecación y prácticas de higiene de manos, así como las muestras de heces de los escolares. Las muestras de heces se examinaron mediante el método de Kato-Katz.
Resultados: Se examinaron muestras de heces de 6,421 escolares. La prevalencia ponderada general de cualquier STH en el estado fue de 75.6 (95 % *IC*: 71.2-79.5). La prevalencia fue mayor al 50 % en seis de las nueve zonas agroclimáticas. *Ascaris lumbricoides* </bx> (*A. lumbricoides*) STH de mayor prevalencia (prevalencia: 69,6 %), seguida del anquilostoma (prevalencia: 22,6 %) y (*Trichuris trichura*) *T. trichura* (4,6 %). La mayoría de las infecciones por STH fueron de baja intensidad. Los factores de riesgo relevantes vinculados a la infección por STH fueron la práctica

de defecación al aire libre y la ausencia de higiene de manos con jabón, y la permanencia en casas kutcha.

Conclusiones: La prevalencia de STH entre los escolares de primaria en Uttar Pradesh fue alta. En base a las directrices de la Organización Mundial de la Salud en cuanto a la frecuencia de desparasitación según la prevalencia de STH, el Gobierno de Uttar Pradesh debe implementar un programa de desparasitación escolar con frecuencia semestral. Los hallazgos de nuestro estudio también ayudarían a controlar el rendimiento del programa de desparasitación escolar.

Translated from English version into Spanish by Diana, through

TRANSLATORS
WITHOUT BORDERS
