# Supplementary material for: High prevalence of soil-transmitted helminth infections among primary school children, Uttar Pradesh, India, 2015
Source: Infect Dis Poverty. 2017 Oct 9;6:139. doi: 10.1186/s40249-017-0354-7 (PMC5632835; doi:10.1186/s40249-017-0354-7)
Supplement: Supplementary file 2 — Agro-climatic zones of Uttar Pradesh and districts sampled. (DOC 37 kb) [file 40249_2017_354_MOESM2_ESM.doc]

**Supplementary Table 1: Agro-climatic zones of Uttar Pradesh and districts sampled**

| **Agro-climatic Zone** | **Population aged 5 – 10 years** | **No. of districts selected** | **No. of schools selected** |
| --- | --- | --- | --- |
| Bhabhar and Tarai | 1 559 902 | Bijnour, Saharanpur | 7 |
| Bundelkhand | 1 442 724 | Banda*, Jhansi | 6 |
| Central | 6 863 523 | Allahabad*, Farrukhabad,* Hardoi*, Kannauj*, Kheri,* Rae Bareli* | 31 |
|  |  |
|  |  |
| Eastern Plain | 5 699 960 | Ghazipur*, Jaunpur*, Mau,* Varanasi* | 24 |
|  |  |
| Mid Western Plain | 2 633 808 | Jyotiba Phule Nagar, Moradabad, Rampur* | 14 |
| North Eastern Plain | 5 980 871 | Balrampur*, Gorakhpur,* Kushinagar*, Siddharthnagar* | 22 |
|  |  |
| South Western Semi Arid | 3 082 927 | Agra, Aligarh, Mathura | 12 |
| Vindhyan | 981 830 | Mirzapur* | 3 |
| Western Plain | 316 3450 | Ghaziabad, Meerut | 11 |
| **Total** | **31 408 995** | **27** | **130** |

(*Lymphatic filariasis endemic districts)
